# The EMA as a potential driver of competitiveness of orphan medicines in Europe: a focus on regulatory incentives and funding opportunities

**DOI:** 10.1186/s13023-025-04095-3

**Published:** 2025-10-28

**Authors:** Maria Ana Gomez-Ferreria, Virginia Garcia-Muñoz, Alex Zwiers

**Affiliations:** Zwiers Regulatory Consultancy, A ProductLife Group Company, Amsterdam, Netherlands

**Keywords:** Orphan drugs, Orphan medicines, Clinical trials, Priority Review Voucher (PRV), Rare Paediatric Disease, Pharmaceutical legislation, European Medicines Agency (EMA), Food and Drug Administration (FDA)

## Abstract

The revision of the European Union (EU) Pharmaceutical Legislation aims for a competitive environment for innovation in the EU, including actions to support the development and approval of orphan medicines. In this article, we look at the Food and Drug Administration (FDA) for references to support this goal and discuss some FDA programs fostering innovation and competitiveness for orphan drugs in the US, including the Priority Review Voucher (PRV) program for Rare Paediatric Diseases, the Orphan Drug Grants to support clinical trials in orphan conditions and the recently launched Rare Disease Evidence Principles (RDEP) process. We further share some thoughts on how similar programs could be implemented in the EU, posing the European Medicines Agency (EMA) as a key figure to improve competitiveness for the development of orphan medicines in Europe.

## Introduction

A main goal of the new European Pharmaceutical Legislation is supporting the innovation capacity and competitiveness of the pharmaceutical sector in the EU, maintaining and reinforcing the position of the EU pharmaceutical industry, both within the EU and globally [1]. In this context, it is acknowledged that an efficient and agile EU regulatory system plays a crucial role in creating a favourable environment for boosting the innovation capacity and competitiveness of the pharmaceutical industry.

Within the whole pharmaceutical sector, the orphan drug market is of high strategical interest, both for its size and its inherent degree of innovation. By 2030, orphan drugs will double their market share to 20% of the $1.6 trillion global drug market, with a Compound Annual Growth Rate (CAGR) of over 10%, outperforming the 7.5% of non-orphan drugs [2, 3]. In line with the strategic value of the orphan drugs market, the new EU Pharma Legislation includes important modifications to the incentives applicable to orphan medicines. The current legal text, still under discussion, proposes the reduction of the duration of market exclusivity from10 years to a 9 years period, or 5 years if registered as a well-established use. However, companies can benefit from additional periods of market exclusivity if they address a high unmet medical need (+ 1 year), launch the medicine in all Member States (+ 1 year), or develop a new therapeutic indication for an already authorised orphan medicine (+ 1 year/indication, up to 2 years). In the US, the market exclusivity period is 7 years, that can be extended 6 months in the case of paediatric indications.

European biotech companies developing orphan medicines often prioritize the US market, which results in a consistently higher number of approvals of orphan medicines in the US each year, compared with Europe [4]. To date, the European Commission (EC) has authorised more than 200 orphan medicines while, in the period 1983–2022, the FDA approved 882 of orphan drugs for use in 392 rare diseases [5, 6]. Even if not strictly necessary, prioritizing the US market leads to prioritizing clinical trials in the US as well, with the consequent loss of value for the EU. Between 2007 and 2022 (15 years), 152 clinical trials were conducted on orphan drugs in the EU, while in the US over 1,000 orphan-designated drugs entered clinical trials in the period 1983–2023 (40 years) [7, 8]. Several factors make development in the US more attractive: larger market size; greater opportunities for funding; adherence to FDA standards for developing highly innovative products; and higher accrual of patients in US hospitals, where just one single regulatory procedure (Investigational New Drug - IND filing) allows recruitment throughout the whole territory.

As regulatory affairs professionals, we cannot influence market size or private funding opportunities. However, we have the responsibility to ensure that the EMA remains strong and competitive to encourage innovation in Europe. This would make the EU more appealing to run clinical trials, benefitting both the patients and the sites involved in the trials. Some specific tools managed by the FDA to support innovation in orphan diseases can definitively serve as an useful reference to take into account in Europe.

## Priority Review Vouchers (PRVs) for Rare Paediatric Diseases in US: a reality

Priority Review is one of the FDA expedited pathways [9]. A Priority Review designation means that the FDA aims to take action on a New Drug Application (NDA) or Biologic Licence Application (BLA) within 6 months, compared to 10 months under standard review. The Priority Review Voucher (PRV) for Rare Paediatric Diseases, also available for tropical diseases (although used to a much lesser extent), has been a major incentive attracting orphan drug developers to the FDA.

Rare Paediatric Disease is a designation granted by the FDA Office of Orphan Product Development (OOPD) to orphan drugs that are used for paediatric populations [10]. Some examples of Rare Paediatric Diseases include: spinal muscular atrophy (SMA), primary hyperoxaluria type 1 (PH1), aromatic L amino acid decarboxylase (AADC) deficiency, or some types of beta-thalassemia, among others. At the time of approval, drugs having a Rare Paediatric Disease designation may be granted a PRV by the FDA. These PRVs can be transferred to another company willing to have its product reviewed under the Priority Review scheme, which otherwise would not benefit from this expedited pathway [10]. Products that have been granted a PRV include, for example: Luxturna (Spark Therapeutics) [11], Zolgensma (AveXis Inc) [12], Oxlumo (Alnylan Pharmaceuticals) [13] or Kebilidi (PTC Therapeutics) [14]. Companies that have purchased and used these vouchers, saving 4 months in the NDA/BLA review process, include Novartis, Gilead or AbbVie, with prices ranging between $21 million and $350 million, and a median price of approximately $100 million. The known highest price of a PRV was $350 million, paid by AbbVie to United Therapeutics in 2015 [15]. United Therapeutics was initially granted this PRV upon approval of unituxin (Qarziba), an antibody indicated for the treatment of some patients with neuroblastoma [16]. This PRV was used by AbbVie for the approval of Rinvoq, a JAK inhibitor initially indicated for the treatment of rheumatoid arthritis [17].

Unfortunately, the FDA PRV program has started to sunset as of 20 December 2024, and so far, no PRVs may be granted beyond 30 September 2026. A similar sunset was set before for September 30, 2020, and the program was then reactivated. However, nowadays, there are no news of the reactivation of this program, and a high degree of uncertainty exists in this regard.

At a glance, the PRV program unravels a smart way of generating value: the US Government provides resources to the FDA to make some extra Priority Reviews; the FDA grants the PRV to companies developing new drugs for rare paediatric conditions; these vouchers are sold to other companies that otherwise could not benefit from Priority Review. Altogether, this program results into a virtuous circle where everyone wins: the company developing the new drug for a rare paediatric disease gets a voucher that can be transferred for millions; the company purchasing the voucher can reach the market 4 months earlier (which could be equivalent to 4 additional months of patent protection); paediatric patients get faster access due to incentivized drug development; and the US Government together with the FDA consolidate the leading role of US in supporting innovation.

## European Accelerated Assessment Vouchers (EAAVs) for rare diseases: a possibility?

Comparable to the FDA Priority Review, the EMA provides Accelerated Assessment, which entails an expedited review of the Marketing Authorization Application (MAA) of a medicinal product expected to be of major public health interest. For standard procedures, the review time of the MAA by the EMA is 210 days, plus two clock stops that allow time for the applicant to respond questions raised by the EMA [18]. The maximum duration of a clock-stop depends on how long the applicant needs to respond, but the first clock-stop usually lasts three months and the second clock-stop one month. Upon a positive opinion of the EMA, the EC will approve the product within 67 days. Adding up these timeframes, the standard procedure for review of the MAA by the EMA and approval by the EC may take around 13 months. For Accelerated Assessments, the EMA review period is reduced to 150 days. The applicants are allowed to have a first clock stop of 1 month and no second clock stop. Also, the EC should provide approval within just 30 days. Adding up these periods, the estimated time for review and approval is reduced to 7 months, being 3–6 months less than the standard procedure (i.e. 6 months if we consider the clock stops). It is fair noting though, that saving time during the clock stops depends on the capacity of the applicant to swiftly respond to the EMA requests.

Similar to the PRV in the US, should we aim for an European Accelerated Assessment Voucher (EAAV)? A thorough analyses of the interest that pharma companies would have in such program, and how much they would be willing to pay for such vouchers would be needed. However, it is tempting to speculate that, even if the high values paid for the FDA PRVs may not be reached, it could still be an incentive for the development of orphan medicines in the EU.

If an EAAV program is implemented, it is also necessary to estimate the resources required by the EMA to manage the additional number of Accelerated Assessments. What could be the impact of such EAAV program for the EMA? While this is hard to predict, we can look at the numbers from the FDA and make a guess. A recent review reports that during 2015–2024, a total of 58 PRVs were granted by the FDA [15], which would account for approximately 6 PRVs per year, even though there is a tendency to higher numbers in the most recent years (9 PRVs in 2023 and 10 PRVs in 2024). It has also been published that, among the 360 approvals granted by the FDA in the period 2015–2022, Priority Review was applied to 65% of the applications [19], thus accounting for approximately 30 Priority Reviews per year. From these numbers, it is possible to estimate that 20% (6 out of 30) Priority Reviews performed annually by the FDA are triggered by the use of PRVs. Therefore, it could be considered that this program meant a 20% increase in the resources needed by the FDA to commit Priority Review. Between 2015 and 2021, the EMA approved a total of 39 products following Accelerated Assessment [20], which accounts for an average of 6 Accelerated Assessments a year, well below the 30 Priority Reviews performed annually by the FDA. In case an EAAV program is created and a higher number of Accelerated Assessments are to be implemented, additional resources should be allocated to the EMA for this purpose, depending on the estimated demand of this program.

Somehow similar, a transferable exclusivity voucher for antimicrobial resistant (AMR) products is being discussed in the context of the new EU Pharmaceutical Legislation [1]. This voucher would grant one additional year of regulatory data protection to the developer of the antimicrobial, which the developer could either use for one of its own products or sell it to another marketing authorization holder (MAH). Such vouchers would create an attractive business case for the development of new antimicrobials, holding the promise of populating the currently limited research pipeline.

## Funding of orphan drugs clinical trials

So far, no financial incentives to support the development of medicinal products (beyond fee reductions for regulatory procedures) are managed by the EMA. Contrarily, the FDA OOPD is responsible for the administration of several grant programs that finance the costs of developing new orphan drugs [21]. Three different programs are currently active, with a budget of up to $22 million in 2024:

- *Clinical Trials Grants Program*. These grants fund clinical trials assessing efficacy and/or safety in rare diseases or conditions. In 2024, the FDA awarded 7 new clinical trials, providing more than $17 million to clinical researchers over 4 years. An active IND is required at the time of submission.
- *Natural History Studies Grants Program*. This program funds well-designed, protocol-driven natural history studies to advance development in rare diseases. In 2024, the FDA funded 3 new grants totalling more than $4.7 million spread over 4 years.
- *Rare Neurodegenerative Disease Grants Program*. This program awards grants and contracts for the development of interventions to prevent, diagnose, or treat amyotrophic lateral disease (ALS) and other rare neurodegenerative diseases.

In the EU, public funds to support clinical trials are managed by the EC, not by the EMA. Horizon Europe (before, Horizon 2020) is the most recent EU funding program [22]. These funds typically support early development clinical trials sponsored by academic institutions or hospitals, and are often awarded to consortiums for various activities beyond the clinical trial. Having specific grants managed by the EMA to support clinical trials in orphan diseases could be an additional incentive for Sponsors to perform clinical trials in Europe. In such case, the EMA would leverage its thorough experience in clinical development and apply its high standards to assess the scientific relevance and feasibility of the clinical trials, same as the FDA is doing when managing the programs outlined above.

In regards to programs funding studies on natural history, there is an strong intention that real-world data (RWD), including natural history, could be used to achieve better informed and more efficient regulatory decision-making [23–26]. Data on natural history for orphan conditions is often the basis for approval when only single arm studies are available. Therefore, in some cases, investing in collecting high quality natural history data should be seen as investing in a control arm for a pivotal study. Given the high level of uncertainty faced when randomized controlled studies are lacking at the time of marketing authorization and health technology assessment (HTA), it would be highly relevant to provide financial support at an EU level to perform these natural history studies. Again, having the EMA involved in the public funding of natural history studies would assure the quality and clinical relevance of such studies.

## Rare Disease Evidence Principles (RDEP) process

In early September, the FDA launched a new process under Rare Disease Evidence Principles (RDEP) to facilitate the approval of drugs to treat rare diseases with very small patient populations (e.g. less than 1,000 persons in the US) with significant unmet medical need and with a known genetic defect that is the major driver of the pathophysiology [27]. While not specifically mentioned in the FDA release note, most of the eligible products will be gene therapies developed for very rare diseases. The RDEP process assures that the drug review will encompass additional supportive data, including case reports, expanded access data or natural history studies. A request should be submitted to an existing IND prior to the launch of the pivotal trial the sponsor wants reviewed under the RDEP. Accepted sponsors will have an initial meeting with the appropriate FDA review team to determine what data will be used to substantiate safety and effectiveness. At this initial meeting, the agency and the sponsor may discuss the need for future engagement. If there is no IND for the product, the sponsor can subsequently open an IND after the meeting.

From our point of view, the most innovative aspect of this process is that it provides support for development in very rare diseases (less than 1,000 patients in the US, compared to the threshold of less than 200,000 patients considered for orphan drug designation). Specific regulatory tools for rare diseases with very small populations are very much needed in both US and Europe, to address the differential hurdles of developing drugs for very rare compared to just rare diseases.

Overall, the regulatory support that can be obtained from the FDA within the RDEP process does not seem to differ much from that provided via the formal meetings with the FDA or the Scientific Advice/Protocol Assistance procedure with the EMA. Indeed, tailored support for programs covering unmet medical needs is already provided in the current FDA expedited programs (RMAT, Breakthrough Therapy, Fast Trak designations) or the EMA PRIME program. However, launching a dedicated process for drugs developed to treat rare diseases with very small patients populations, having an IND, will likely result in making the US landscape even more attractive for developers. The generation of similar tools in Europe, designed and managed by the EMA, should be considered.

## Conclusion

The new EU Pharmaceutical Legislation provides considerations to foster innovation and make the European landscape more competitive for the development of innovative orphan medicines. Beyond streamlining regulatory procedures and providing additional incentives on market exclusivity, it could be worth considering the implementation of specific programs that provide additional financial incentives and grants to support clinical development of orphan medicines in Europe. Consideration to FDA programs like the PRV, the Orphan Drug Grants or the RDEP process should be given by the different stakeholders. Tools available in the US rely on the thorough expertise of the FDA, consolidating the leading role of the US regulatory agency in fostering innovation. Providing the EMA with similar resources and responsibilities could result in having a more attractive space for the development of orphan products in Europe, retaining the high value of performing clinical trials in this territory and providing faster access to the patients in need. 

Overall, beyond the measures under discussion in the new EU Pharmaceutical Legislation, we believe that the EMA should play a major role in improving the competitiveness of Europe for the development and approval of orphan medicines. Endorsing the EMA with additional resources and responsibilities has the potential to retain and bring new developers of orphan medicines. This would increase clinical trial activity, market approval and availability of orphan medicines across Member States.

## Data Availability

Not applicable.

## Abbreviations

BLA: Biologic Licence Application
EAAV: European Accelerated Assessment Voucher
EC: European Commission
EMA: European Medicines Agency
EU: European Union
FDA: Food and Drug Administration
IND: Investigational New Drug
MAA: Marketing Authorization Application
NDA: New Drug Application
OOPD: Office of Orphan Product Development
PRV: Priority Review Voucher
RDEP: Rare Disease Evidence Principles

## References

[1] COM(2023) 190 final. Communication from the commission to the European Parliament, the Council, the European Economic and Social Committee and the Committee of the regions - Reform of the pharmaceutical legislation and measures addressing antimicrobial resistance. 2023. Available from: https://eur-lex.europa.eu/legal-content/EN/TXT/PDF/?uri=CELEX:52023DC0190. Accessed 16 May 2025.

[2] Spencer D. Orphan drugs will be a fifth of prescription drug sales by 2030. News, Trends & Analysis. 2025. Available from: https://eur-lex.europa.eu/legal-content/EN/TXT/PDF/?uri=CELEX:52023DC0190. Accessed 16 May 2025.

[3] Kaylor A. Orphan drugs will double their market share by 2030. TechTarget - Xtelligent Pharma Life Sciences. 2025. Available from: https://www.techtarget.com/pharmalifesciences/news/366621763/Orphan-drugs-will-double-their-market-share-by-2030#:~:text=By%25202030%252C%2520orphan%2520drugs%2520will,%2525%2520of%2520non%252Dorphan%2520drugs.%26text=Once%2520specialized%2520treatments%2520for%2520rare,the%2520dominance%2520of%2520mainstream%2520medications. Accessed 16 May 2025.

[4] Giannuzzi V, et al. Orphan medicinal products in Europe and United States to cover needs of patients with rare diseases: an increased common effort is to be foreseen. Orphanet J Rare Dis. 2017;12(1):64. Available from: 10.1186/s13023-017-0617-1. 10.1186/s13023-017-0617-1 PMC5376695 28372595

[5] European Commission. Orphan medicinal products. 2025. Available from: https://health.ec.europa.eu/medicinal-products/orphan-medicinal-products_en. Accessed 12 September 2025.

[6] Fermaglich LJ, Miller KL. A comprehensive study of the rare diseases and conditions targeted by orphan drug designations and approvals over the Forty years of the orphan drug act. Orphanet J Rare Dis. 2023;18(1):163. 10.1186/s13023-023-02790-7. 37353796 10.1186/s13023-023-02790-7 PMC10290406

[7] Mishra S, Venkatesh MP. Rare disease clinical trials in the European union: navigating regulatory and clinical challenges. Orphanet J Rare Dis. 2024;19(1):285. 10.1186/s13023-024-03146-5. 39085891 10.1186/s13023-024-03146-5 PMC11292868

[8] Yahoo Reports. Over 1000 orphan drugs in clinical trials in the US - Eylea, Actimmune, Ravicti Among Key Approved Therapies. 2023. Available from: https://finance.yahoo.com/news/over-1000-orphan-drugs-clinical-104300470.html. Accessed 12 September 2025.

[9] CDER and CBER (FDA), Expedited programs for serious conditions – drugs and biologics - guidance for industry. 2014. Available from: https://www.fda.gov/media/122425/download

[10] FDA. Rare pediatric disease designation and priority review voucher programs. 2024. Available from: https://www.fda.gov/industry/medical-products-rare-diseases-and-conditions/rare-pediatric-disease-designation-and-priority-review-voucher-programs. Accessed 12 May 2025.

[11] CBER (FDA). Approval letter - Luxturna (voretigene neparvovec-rzyl). 2017. Available from: https://www.fda.gov/vaccines-blood-biologics/cellular-gene-therapy-products/luxturna. Accessed 10 September 2025.

[12] CBER (FDA). Approval Letter - Zolgensma (vonasemnogene abeparvovec-xioi). 2019. Available from: https://www.fda.gov/vaccines-blood-biologics/zolgensma. Accessed 10 September 2025.

[13] CBER (FDA). Approval Letter - Oxlumo (lumasiran). 2020. Available from: https://www.accessdata.fda.gov/drugsatfda_docs/nda/2020/214103Orig1s000Approv.pdf. Accessed 10 September 2025.

[14] CBER (FDA). Approval Letter - Kebilidi (eladocagene exuparvovec-tneq). 2024. Available from: https://www.fda.gov/vaccines-blood-biologics/kebilidi. Accessed 10 September 2025.

[15] Armstrong A. Priority review vouchers: by the numbers. BioSpace 2025. Available from: https://www.biospace.com/business/priority-review-vouchers-by-the-numbers. Accessed 16 May 2025.

[16] Label, Unituxin. Unituxin^®^ (dinutuximab) injection, for intravenous use. 2020. Available from: https://www.accessdata.fda.gov/drugsatfda_docs/label/2020/125516s025lbl.pdf

[17] Label, Rinvoq. Rinvoq^®^ (upadacitinib) extended-release tablets, for oral use. 2023. Available from: https://www.accessdata.fda.gov/drugsatfda_docs/label/2023/211675s015lbl.pdf

[18] EMA/821278/2015, European Medicines Agency pre-authorisation procedural advice for users of the centralised procedure. 2025. Available from: https://www.ema.europa.eu/en/documents/regulatory-procedural-guideline/european-medicines-agency-pre-authorisation-procedural-advice-users-centralised-procedure_en.pdf

[19] Ronnebaum S, Andrawes M, Smith D. FDA expedited review programs on new drug approval time. Evidera - PPD 2024. Available from: https://www.ispor.org/docs/default-source/intl2024/ispor24ronnebaump28presentation138089-pdf.pdf?sfvrsn=2a1d2da_0. Accessed 16 May 2025.

[20] Vallano A, Pontes C, Agustí A. The challenges of access to innovative medicines with limited evidence in the European union. Front Pharmacol. 2023;14:1215431. 10.3389/fphar.2023.1215431. 37719853 10.3389/fphar.2023.1215431 PMC10500193

[21] FDA Office of Orphan Products Development (FDA). Orphan products grants program. 2025; Available from: https://www.fda.gov/industry/grant-programs-support-development-medical-products-rare-diseases/orphan-products-grants-program. Accessed 12 May 2025.

[22] European Commission. Horizon Europe. 2025; Available from: https://research-and-innovation.ec.europa.eu/funding/funding-opportunities/funding-programmes-and-open-calls/horizon-europe_en. Accessed 16 May 2025.

[23] CDER and CBER (FDA), Rare Diseases: natural history studies for drug development - draft guidance for industry. 2023. Available from: https://www.fda.gov/media/122425/download

[24] EMA/152628/2024, Real-world evidence provided by EMA - Support for regulatory decision-making. 2024. Available from: https://www.ema.europa.eu/en/documents/other/guide-real-world-evidence-provided-ema-support-regulatory-decision-making_en.pdf

[25] EMA/426390/2021, Guideline on registry-based studies. 2021,. 2021. Available from: https://www.ema.europa.eu/en/documents/scientific-guideline/guideline-registry-based-studies_en.pdf

[26] Giannuzzi V, Stoyanova-Beninska V, Hivert V. Editorial: the use of real world data for regulatory purposes in the rare diseases setting. Frontiers in Phar. 2022;13–2022. Available from: https://www.frontiersin.org/journals/pharmacology/articles/10.3389/fphar.2022.1089033. 10.3389/fphar.2022.1089033 10.3389/fphar.2022.1089033 PMC9730868 36506540

[27] CDER/CBER (FDA). Rare Disease Evidence Principles (RDEP) Process. 2024; Available from: https://www.fda.gov/industry/fda-rare-disease-innovation-hub/cdercber-rare-disease-evidence-principles-rdep. Accessed 12/09/2025.

